# Redox potential driven aeration during very-high-gravity ethanol fermentation by using flocculating yeast

**DOI:** 10.1038/srep25763

**Published:** 2016-05-10

**Authors:** Chen-Guang Liu, Xue-Mi Hao, Yen-Han Lin, Feng-Wu Bai

**Affiliations:** 1 School of Life Sciences and Biotechnology, Shanghai Jiao Tong University, Shanghai 200240, China; 2 School of Life Science and Biotechnology, Dalian University of Technology, Dalian 116023, China; 3 Department of Chemical and Biological Engineering, University of Saskatchewan, Saskatoon SK S7N 5A9, Canada

## Abstract

Ethanol fermentation requires oxygen to maintain high biomass and cell viability, especially under very-high-gravity (VHG) condition. In this work, fermentation redox potential (ORP) was applied to drive the aeration process at low dissolved oxygen (DO) levels, which is infeasible to be regulated by a DO sensor. The performance and characteristics of flocculating yeast grown under 300 and 260 g glucose/L conditions were subjected to various aeration strategies including: no aeration; controlled aeration at −150, −100 and −50 mV levels; and constant aeration at 0.05 and 0.2 vvm. The results showed that anaerobic fermentation produced the least ethanol and had the highest residual glucose after 72 h of fermentation. Controlled aerations, depending on the real-time oxygen demand, led to higher cell viability than the no-aeration counterpart. Constant aeration triggered a quick biomass formation, and fast glucose utilization. However, over aeration at 0.2 vvm caused a reduction of final ethanol concentration. The controlled aeration driven by ORP under VHG conditions resulted in the best fermentation performance. Moreover, the controlled aeration could enhance yeast flocculating activity, promote an increase of flocs size, and accelerate yeast separation near the end of fermentation.

Although there is controversy regarding fuel ethanol production by fermenting starch-based feedstock, which can otherwise be consumed by humans, there is no doubt that ethanol is the most utilized bio-energy all around the world^1^. To reduce the dependence on sugar or starch-based feedstock, lignocellulosic biomass such as agriculture residue and forestry waste should be used as the new raw material for ethanol fermentation. Unfortunately, the objectives of pretreatment with low energy consumption, high efficiency cellulose production, and constructing ideal strains with high stress tolerance and the ability to ferment pentose are still not able to satisfy industrial needs.

Reduction of energy demand during ethanol fermentation is one of the objectives in the fuel alcohol industry. Very-high-gravity (VHG) fermentation with initial glucose concentration greater than 250 g/L can significantly save energy costs during the distillation and the wastewater treatment stage of operation^2^,^3^. In addition, the use of flocculating yeast can also lower operating costs when separating yeast aggregates (aka, flocs) from the fermentation broth^2^. The formation of yeast flocs helps this yeast strain enduring ethanol toxicity, which makes it suitable to propagate under VHG conditions^4^.

However, the stressful environment encompassing VHG fermentation results in incomplete glucose utlization at the end of fermentation (aka, stuck fermentation), leading to slow yeast growth and low yeast viabilty. Oxygen helps yeast reinforce its stress tolerance through systhesis of sterols and unsaturate fatty acids involved in maintaining membrane health^5^. Oxygen also promotes cell regeneration through TCA cycle and respiration pathway by re-fluxing essential skeletons during bioenergic synthesis and carbon utilization.

Converting glucose to ethanol is a redox neutral process in yeast where the involvement of molecular oxygen may not be necessary. However, due to the complexity of intertwining intracellular bionetwork with extracellular environment, yeast does not follow a simple equation. Thus, supplementation of oxygen becomes a common approach to improve ethanol production during fermentation^6^. Unfortunately, viable cells usually consume oxygen in the medium much faster than the dissolution of oxygen from air even under a large sparging flow rate, it thus leads to the very low levels of dissolved oxygen which is infeasible to monitor with a conventional oxygen electrode during the fermentation process. To quantify the presence of trace amounts of dissolved oxygen, a redox potential (aka, ORP) sensor is used due to its high sensitivity to half-cell reaction O_2_/H_2_O, which has the highest stardard redox potential among the many normal cell metabolites^7^,^8^. As a matter of fact, the dissolved oxygen sensor is a type of ORP sensor except that the sensor tip is covered by an oxygen-permeable membrane. Controlling ORP during fermentation has been verified as an effective strategy to alter metabolic flux distribution towards desired metabolic products^9^,^10^,^11^,^12^. Although some work has been done to demostrate the effect of ORP on ethanol fermentation under limited oxygen environment^13^,^14^,^15^,^16^, the effect of a full spectrum of oxygen level, from no aeration to fully aerated, on flocculating yeast during ethanol fermentation is lacking.

The aeration conditions used in this communication inlcude: no aeration, controlled aeration by altering fermentation ORP level (−150, −100, −50 mV), and constant aeration at 0.05 and 0.2 vvm.

## Results and Discussion

### Aeration conditions and ORP profiles

**Figure 1:**
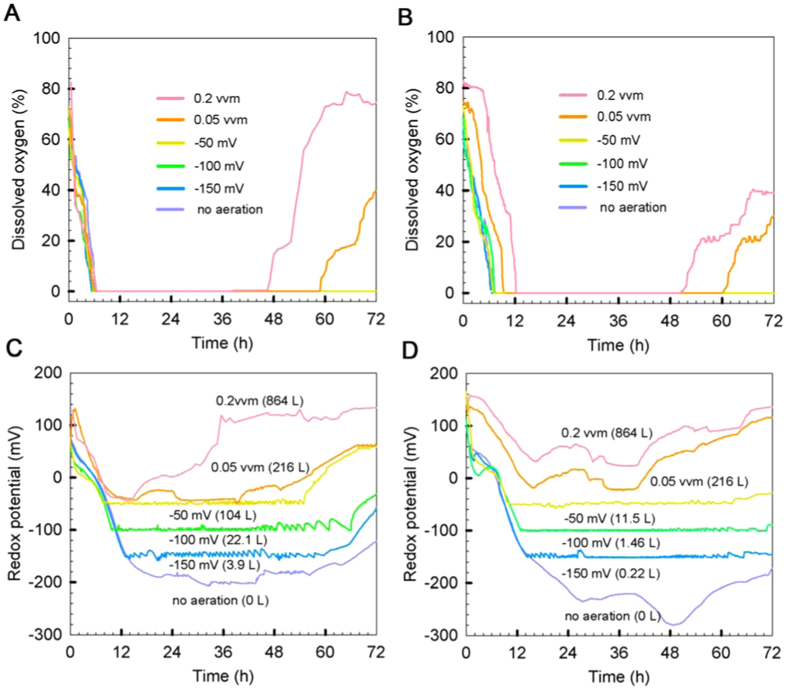
Profiles of dissolved oxygen (**A,B**) and redox potential (**C,D**) under various aeration conditions for initial glucose 260 g/L (**A,C**) and 300 g/L (**B,D**). The number shown in parentheses indicates the total air supplied for a 72-h fermentation.

As shown in Fig. 1A,B, the DO level could only be detected at the initial stage of fermentation under all investigated aeration conditions and near the end of fermentation under constant aeration. For most of the fermentation time, the DO reading was nearly zero because the consumption of DO by viable yeast was much faster than the oxygen dissolution process from air to liquid phase even at the large sparging rate such as 0.2 vvm. Therefore, it is very difficult to distinguish all aeration strategies in the view of DO profiles.

In contrast, ORP profiles by flocculating yeast grown under various aeration conditions are illustrated in Fig. 1C,D. A typical bathtub-shaped profile, as previously reported by Lin *et al.*^11^,^13^, was observed irrespective of various aeration conditions and glucose concentrations. It is noticed that the ORP profiles were elevated as air supplementation was increased during the course of fermentation. However, the air supplementation is not the sole factor to govern ORP profiles. Under the same air flow rate at 0.05 vvm, the ORP level under 300 g glucose/L condition resulted in higher ORP level than that under 260 g glucose/L condition. This indicates that yeast cells were relatively inactive due to osmotic stress induced by high sugar concentration (Fig. 1D). On the other hand, less osmotic pressure was imposed to yeast cells, thereby a relatively high cell viability resulting in reducing power that outweighs oxidizing power (Fig. 1C). As a consequence, a relatively low ORP profile (Fig. 1C vs. Fig. 1D) was observed under all constant aeration conditions (ie, 0.05 and 0.2 vvm).

It has been reported that ORP profile is tightly correlated to yeast growth^11^. When yeast begins to propagate irrespective of aeration conditions, the demand of oxygen consumption increases, resulting in a decline of ORP profile during this stage of growth. As proceeding to mid-exponential phase, the build-up of reducing metabolites causes a steep descending in ORP profile for all investigated aeration cases except constant aeration at 0.05 and 0.2 vvm.

Controlled aeration resulted in a basin found in the bathtub-like ORP profile, which realizes a proper oxygen supply matching with demand by yeast in order to reshuffle reducing equivalents such as NADH/NAD^+^ pairs. Cases of constant aeration and no aeration have the most similar profiles; however, constant aeration elevates the profiles, which reflect accelerated growth promoted by oxygen supply.

### Yeast growth and viability under various aeration conditions

**Figure 2:**
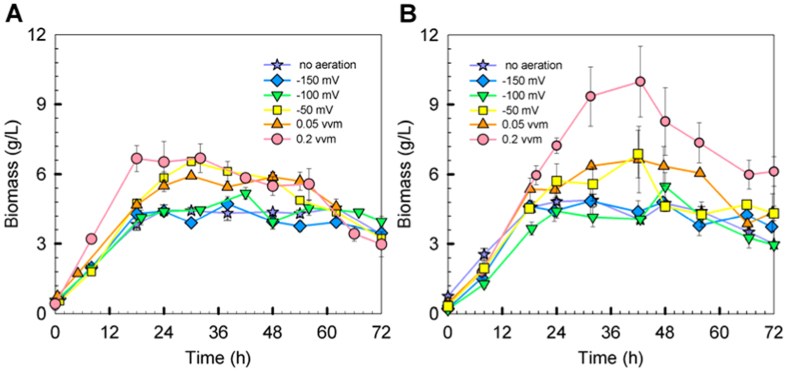
Biomass under various aeration conditions for initial glucose (**A**) 260 g/L and (**B**) 300 g/L.

**Figure 3:**
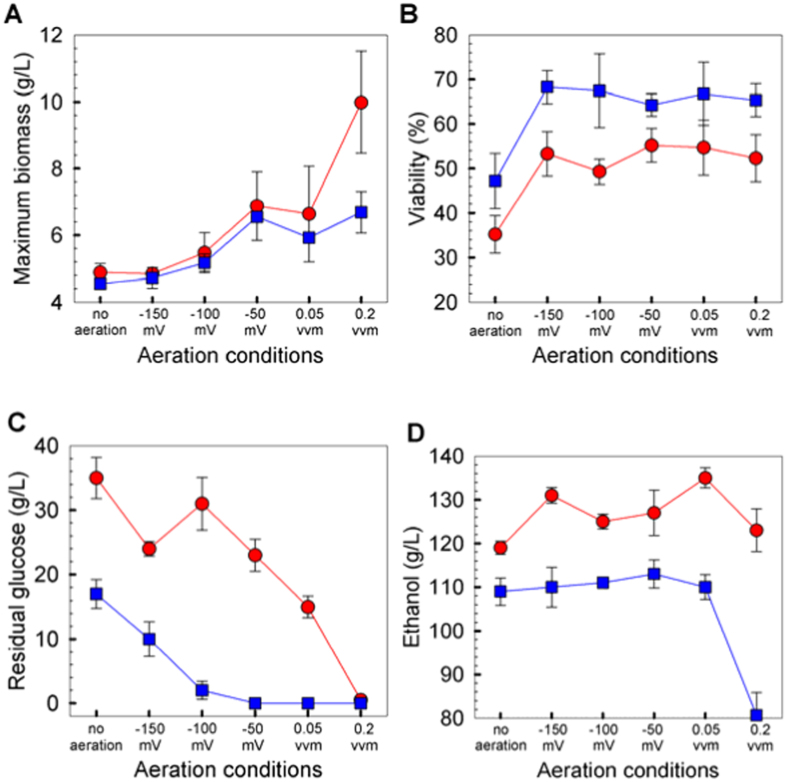
Effects of total air supplied on (**A**) maximum biomass (**B**) cells viability, (**C**) residual glucose and (**D**) final ethanol concentration after 72 h fermentation. Blue symbol for initial glucose at 260 g/L and red for 300 g/L.

In reference to Fig. 2, the biomass concentration profile under controlled aeration condition is similar to that of no aeration counterpart except for the condition where ORP was controlled at −50 mV (Fig. 2). In this case, the maximum biomass concentration was about 50% higher than that without aeration. Aeration promotes biomass formation (Fig. 1C, aeration vs. no aeration). More importantly, aeration extends the exponential phase of yeast during ethanol fermentation (Fig. 2). As shown in Fig. 3A, when the air supply changed from no aeration to aeration condition, the maximum biomass increased accordingly, and the better maximum biomass could be seen under 300 g glucose/L condition. Oxygen is not only helping cell to construct healthier membranes via synthesis of sterols and unsaturated fatty acids^5^, but also triggering the respiratory route to energize cell regeneration. Although formation of biomass will compete with the desired product for carbon source, a higher biomass will benefit rapid ethanol production as ethanol is the primary metabolite for yeast.

Figure 3B illustrated aeration controlled at −150 mV level increases cell viability from no aeration counterpart by 44.7% (with 3.9 L total air supply) and 51.4% (with 0.22 L total air supply) for 260 g glucose/L and 300 g glucose/L case, respectively. However, the viability remained stable with the increase in total air supply when the redox potential level was above −150 mV. This observation indicates that it is oxygen present, but not the oxygen amount in the air stream, that contributes to cell viability. Although higher initial glucose concentration imposes more severe osmotic stress to yeast growth and viability, more effective improvement in cell viability by aeration was evident under higher glucose concentration. For example, under constant aeration at 0.05 vvm with 216 L total air supply, compared to no aeration fermentation, cell viability increases by 48.6% for 300 g glucose/L case, which was higher than the 41.5% for 260 g glucose/L case.

### Overall performance under various aeration conditions

The rate of substrate utilization is affected by biomass concentration and cell viability. A trace amount of oxygen plays a pivotal role in modulating yeast viability, and the presence of more oxygen would accelerate yeast propagation, resulting in low residual glucose concentration (Fig. 3C). Excessive amounts of under-utilized glucose is unacceptable in the industrial setting because of the cost of raw material and the energy consumption during downstream processing. Thus, integration of fermenter configuration and air supply could be applied to enhance substrate conversion^17^,^18^. Under 300 g glucose/L condition, it is noteworthy to mention that the residual glucose under −150 mV control was lower than that without aeration and that under ORP control at −100 mV. This implies that the choice of ORP control level might be more crucial than total air supply.

Ethanol level is of essential importance in VHG ethanol fermentation due to product inhibition. Proper aeration would accelerate yeast to build up population and maintain it at high yeast viability, resulting in shorter fermentation time. Generally, ethanol concentration profile shows an inverse relationship to glucose counterpart (Fig. 3D). However, too much air supplementation would enhance the air stripping effect, resulting in ethanol loss. On the other hand, the presence of oxygen alters carbon re-distribution to respiratory pathway from fermentative route, thereby lowering ethanol production. This indicates that there exists an optimal aeration condition where a maximum ethanol concentration is attainable during fermentation.

Yeast cells in different growth phases have a specific demand for oxygen. For example, growing at the exponential phase requires more oxygen than that at the death phase. Constant aeration leads to oxygen shortage during rapid growth phase and excess during lag and death phase. Moreover, excess oxygen brings the risk of contamination and loses ethanol through air stripping. Therefore, controlling aeration based on fermentation ORP reading would result in the highest ethanol productivity.

### Yeast flocculation under various aeration conditions

**Figure 4:**
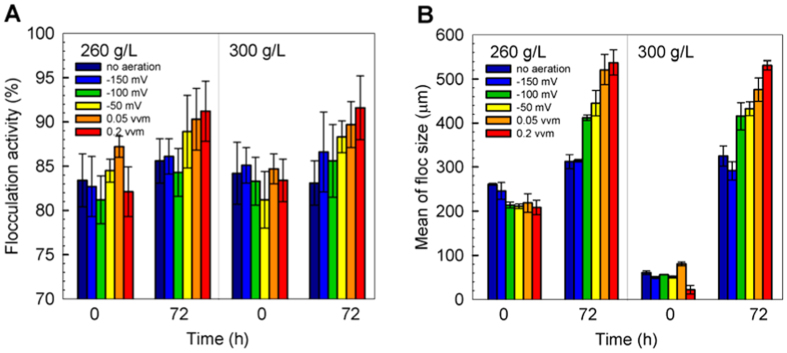
Comparison of flocculating characteristic under different aeration conditions. (**A**) flocculation activity, and (**B**) mean flocs size during ethanol fermentation.

Yeast flocculation activity measured at the beginning and at the end of fermentation was compared (Fig. 4A). After 72 h fermentation, the yeast flocculation activity under all investigated conditions were enhanced. The increase of flocculation activity under constant aeration is remarkable, probably owing to the presence of oxygen where the cell surface became modified via the interaction between lectin and mannose^4^. Thus the quantity and activity of glycoprotein predominates the flocculating characteristic at molecular level, where the synthesis of glycoprotein is related to the oxygen availability.

*In situ* flocculation size distribution by FBRM was monitored during the course of fermentation. The FBRM relates various environmental factors including broth behavior, temperature, pH, biomass, and media compositions to flocs size of yeast. Figure 4B illustrates the average size distribution of flocs between 0 and 72 h of fermentation. The increase of flocs size at the end of fermentation was not only causing by the enhanced flocculation activity, but also resulting from the increased biomass and ethanol concentration. Irrespective of initial glucose concentration, constant aeration leads to higher biomass concentration (Fig. 2), providing yeast cells more contact surface to form larger flocs.

## Conclusions

This work investigated the effects of various aeration strategies on VHG ethanol fermentation and yeast flocculating characteristics by using an ORP sensor instead of a traditional DO probe. Based on the cell growth and ORP profiles, the ORP reading below −200 mV is deemed as anaerobic fermentation where low yeast viability and low biomass was recorded. In contrast, above −50 mV is regarded as aerobic fermentation where high yeast viability and significant increase of biomass was observed. Controlling ORP level between −200 and −50 mV could be called micro-aerobic fermentation. More air supply promotes biomass formation and enhances yeast viability, accelerating glucose utilization and ethanol production. However, over supplementation of air such as under 0.2 vvm condition, stripped off ethanol, consequently lowering final product concentration and product yield. The optimal aeration scheme would be ORP-controlled fermentation, featuring accurate oxygen supply in order to meet the need for yeast during the course of fermentation and to maintain a stable redox environment to achieve high ethanol yield. The flocculating activity was improved with the increase of total air supply as well.

## Materials and Methods

### Strain and fermentation

The flocculating yeast strain (*Saccharomyces cerevisiae* SPSC01) used in this work was bred by authors’ lab in Dalian University of Technology^19^. Yeast cells were pre-cultured in media containing (g/L): yeast extract, 4; peptone, 3; dextrose, 30; for 18 h in a shake flasks with 100 mL working volume, and then inoculated (5%) to fermenter with 1 L working volume that hold YPD medium, containing (g/L) dextrose, either 300 or 260. Temperature, pH, agitation rate and fermentation time were kept at 30 °C, 4.5, 150 rpm and 72 h, respectively. Two 4-mL aliquots were sampled from the fermenter every 6 or 8 h, one for metabolite quantification and another for determining degree of yeast flocculation. All fermentations were carried out in duplicate.

### Aeration conditions

No aeration means that no air was sparged during the course of fermentation. Controlled aeration means that membrane-sterile air was supplemented according to pre-set ORP level: −150, −100, or −50 mV. Fermentation ORP control has been reported previously^11^. Briefly, an ORP sensor (Pt4805-DPAS-SC-K8S/225, Mettler Toledo, Switzerland) was used to measure fermentation ORP level which was then processed by an ORP controller (model: Transmitter PC-3100, Suntex, Taiwan) where an air pump and a 0.2 μm air filter were connected to. As the measured ORP level is lower than the set point, the air pump is activated by the ORP controller. When the set point is attained, the pump is then turned off. Constant aeration means that a stream of membrane-sterile air was sparged into fermenter at 0.05 or 0.2 vvm. The fermentation DO reading was recorded by using a DO sensor (InPro-6820, Mettler Toledo, Switzerland) for all aeration conditions.

### Sample analysis

Yeast viability was obtained by calculating the ratio of live cells to total cells via methylene blue staining procedure^20^. Biomass was determined by gravimetric method where samples were centrifuged (9000 × g, 4 °C) for 5 min, washed twice with pure water, dried at 85 °C for 24 h, and weighed. The supernatant was then used to quantify glucose and ethanol by HPLC (Model: Waters 1525) with an ion exclusion column (Model: Aminex HPX-87 H 300 × 7.8 mm, Bio-Rad, US) and an RI detector (Model: Waters 2414). The mobile phase consisted of 0.01 mol/L H_2_SO_4_, and the flow rate was set at 0.6 mL/min. The temperature for column and RI detector both were kept at 50 °C.

### Measurement of degree of flocculation

Yeast cells were de-flocculated with 0.1 mol/L EDTA for 1 min, washed twice and then suspended in deionized water. Calcium chloride (0.3 mL of 0.1 mol/L) was added into 3 mL cell suspension whose initial absorbance (AB_a_) was measured at 650 nm. Cell suspension was vortexed for 30 s, settled for 3 min, and the final absorbance was taken (AB_b_). Flocculating activity (also called intrinsic flocculating ability) was then calculated using the following equation:


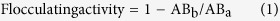


A beam reflectance measurement system (FBRM, Mettler Toledo, Switzerland) was inserted into the fermenter to monitor *in situ* flocs size distributions, and the mean size of flocs was calculated as apparent flocculating ability^21^.

## Additional Information

**How to cite this article**: Liu, C.-G. *et al.* Redox potential driven aeration during very-high-gravity ethanol fermentation by using flocculating yeast. *Sci. Rep.* **6**, 25763; doi: 10.1038/srep25763 (2016).
