# Corrigendum: Redox potential driven aeration during very-high-gravity ethanol fermentation by using flocculating yeast

**DOI:** 10.1038/srep30995

**Published:** 2016-08-25

**Authors:** Chen-Guang Liu, Xue-Mi Hao, Yen-Han Lin, Feng-Wu Bai

Scientific Reports 6: Article number: 25763; 10.1038/srep25763published online: 05 10 2016; updated: 08 25 2016

In the original version of this Article, Affiliation 1 was incomplete. The correct affiliation is listed below:

State Key Laboratory of Microbial Metabolism and School of Life Sciences and Biotechnology, Shanghai Jiao Tong University, Shanghai 200240, China.

This error has now been corrected in the PDF and HTML versions of the Article.

